# Neural Correlates of Mentalizing Altered in Childhood Trauma and Cocaine Use Disorder

**DOI:** 10.21203/rs.3.rs-8407565/v1

**Published:** 2026-01-09

**Authors:** Gabrielle Aude Zbären, Philip Kamilar-Britt, Vyoma Sahani, Rebecca Schusterman, Scott J. Moeller, Vilma Gabbay, Yasmin Hurd, Keren Bachi

**Affiliations:** 1 Department of Psychiatry, Icahn School of Medicine at Mount Sinai, New York, NY; 2 Department of Psychology, University at Albany, State University of New York, Albany, NY; 3 Department of Psychiatry and Behavioral Health, Renaissance School of Medicine at Stony Brook University, Stony Brook, NY; 4 Department of Psychiatry and Behavioral Sciences, The University of Miami Miller School of Medicine, Miami, FL; 5 Nathan Kline Institute for Psychiatric Research, Orangeburg, NY; 6 Department of Environmental Medicine, Icahn School of Medicine at Mount Sinai, New York, NY

**Keywords:** Cocaine use disorder, Childhood trauma, Mentalizing, fMRI, Social functioning, precuneus

## Abstract

**Background:**

Childhood trauma is highly prevalent among individuals with cocaine use disorder (iCUD), and both entail social cognition deficits, including impaired mentalizing (social inference) capacity. Here we sought to examine the neuro-circuits underlying these associations using task-based neuroimaging. We hypothesized that childhood trauma and CUD would show altered brain activation of the mentalizing network, related to deficits in real-world social capacities.

**Methods:**

Participants (45 iCUD and 34 healthy controls (HC), with high/low trauma) performed the validated Why/How fMRI task, probing *Why* versus *How* photographed naturalistic behaviors are performed. Whole-brain analyses used a Why > How contrast at the first level, followed by group comparisons at the second level, with cluster-level family-wise error correction (p_FWE_ < .05). Social functioning and clinical measures were obtained using validated self-report instruments.

**Results:**

Mentalizing task behavior outcomes were worse in iCUD than HC (F_(1,75)_ = 4.45, p < .05), with no effect of trauma severity on accuracy. A significant interaction was observed in the precuneus, with greater BOLD responses in iCUD-low than HC-low, and lower responses in iCUD-high than HC-high. High-trauma individuals showed increased frontal pole activation, correlating positively with mentalizing accuracy (r_(39)_ >= .24, p < .05) and social anxiety (r_(79)_ = .29, p = .01), and negatively with resilience (r_(39)_ <= −.37, p < .001).

**Conclusion:**

Greater frontal activation in high-trauma individuals may support task accuracy but is linked to poorer real-world social functioning. Additionally, the link between CUD diagnosis and precuneus activity depends on trauma severity, offering neural insights into how trauma history may influence CUD and social function.

## 1. Introduction

Individuals with cocaine use disorder (iCUD) frequently experience social stress, such as childhood maltreatment, which may shape neural and physiological responses to social interactions and increase vulnerability to poor clinical outcomes. Childhood trauma is highly prevalent in this population^1^ and has been linked to more severe substance use patterns, including increased cocaine consumption, polysubstance use, higher risk of relapse, and heightened responses to drug cues^2 4^. A dose-response relationship between exposure to multiple traumas and cocaine use has been observed^5^. Despite its broad clinical significance, the neurobiological underpinnings of the long-term effects of childhood trauma in CUD remain poorly understood.

Both childhood trauma and CUD are linked to a heightened propensity for impairments in social cognition, particularly in mentalizing - the ability to infer other’s thoughts, intentions, and emotions. Mentalizing is key for clinical trajectory as it is fundamental to forming trusting supportive relationships and promotes adaptive responses to evolving social dynamics. Individuals with a history of childhood trauma are more likely to experience difficulties accurately attributing mental states to others, with evidence suggesting that mentalizing abilities diminish further with each additional type of childhood trauma experienced^6–8^. Individuals with CUD exhibit similar deficits in mentalizing capacity, which has been implicated as a core symptom of substance use disorders^9,10^. Chronic cocaine use has been associated with impaired emotional empathy and diminished mental perspective taking compared to stimulant-naïve populations^11^, and mentalizing deficits correlated with greater craving and a higher number of hospitalizations^12^. Understanding the neural mechanisms underlying social dysfunction, a core phenomenology of CUD, is important to complement current knowledge of CUD as a brain disorder.

The brain regions engaged during mentalizing in healthy individuals - the prefrontal cortex, superior temporal sulcus, temporoparietal junction, and precuneus - form a network that supports social inference by representing the affective and cognitive mental states of the self and others^13^. Some of these regions are implicated in the pathophysiology of substance use disorders, in relation to impaired decision-making, emotion regulation, and social functioning^14–17^. Alterations in this network have also been shown in individuals with a history of childhood trauma^18,19^. Together, the convergence of symptoms and the overlap between the mentalizing network and regions altered in both CUD and childhood trauma point to this network as a potential neural pathway linking childhood trauma to social-cognitive deficits that predict and sustain substance use disorder. Our study aims to characterize the shared and distinct neural underpinnings of mentalizing deficits in individuals with CUD and a history of childhood trauma. Given the critical role of mentalizing in healthy social functioning, elucidating the unique contribution of CUD and childhood trauma to the behavioral, neural, and clinical alterations associated with impaired mentalizing may facilitate the development of targeted evidence-based treatments.

Here, we sought to examine the separate and joint contributions of childhood trauma and CUD on the neural correlates of mentalizing using functional magnetic resonance imaging (fMRI) during a validated mentalizing task. Participants with and without CUD were stratified by childhood trauma severity. We also assessed real-world social functioning to investigate how alterations in mentalizing-related neural activity relates to clinically relevant outcomes.

## 2. Materials and methods

### 2.1 Participants

Study participants (N=96) underwent functional MRI while performing a mentalizing task and completed procedures for clinical and behavioral phenotyping. This study was approved by the Institutional Board of the Icahn School of Medicine at Mount Sinai. All participants provided written informed consent before participation and received monetary compensation upon completion. **Inclusion criteria for all participants were as follows:** ability to understand and give informed consent, age between 18 and 60 years, right-handedness, native English proficiency, and good health. **For iCUD, inclusion criteria were:** current DSM-5 diagnosis for cocaine use disorder and at least a 12-month history of cocaine dependence. **Exclusion criteria for all participants were:** current dependence on alcohol, opioids or other drugs except for nicotine or caffeine, and cocaine for iCUD, urine positive for any psychoactive drugs except cocaine for iCUD, head trauma with loss of consciousness of more than 30 minutes, present or past history of neurological disease of central origin (including seizures) or a DSM-5 psychiatric disorder (including panic attacks or psychosis), except for disorders of high comorbidity with cocaine addiction (e.g., depression), cardiovascular disease (including high blood pressure), any other medical conditions that may alter cerebral function, specifically, endocrinological (including metabolic), oncological, or autoimmune diseases for which the participant is required to take medications, current pregnancy or breast feeding, and contraindications to the MRI environment (e.g., claustrophobia, metal implants).

### 2.2 Clinical and behavioral phenotyping: Clinical, cognitive, and social measures

#### 2.2.1 Childhood trauma assessment

Childhood trauma was measured using the Childhood Trauma Questionnaire (CTQ)^20^, which assesses five types of childhood maltreatment on a 5-point Likert type scale of severity, i.e., sexual, physical, and emotional abuse, and emotional and physical neglect, on a 5-point Likert type scale of severity. Each of the five CTQ subscales has a possible score range of 5 to 25, while the total CTQ score, reflecting the combined score of all subtypes, ranges from 25 to 125. Due to the high prevalence of co-occurring maltreatment types^21^, and the association of exposure to multiple types of trauma with poor health outcomes^7,22,23^, we used the CTQ total score to classify participants into low vs. high trauma groups (see section 2.7.1 for details).

#### 2.2.2 Demographic and substance use data

Urine toxicology and breathalyzer tests were conducted during participant screening and on the day of the fMRI visit to assess recent substance use. Lifetime substance use (in years) for nicotine, alcohol, cannabis, and cocaine, along with years of formal education, were assessed using the Addiction Severity Index^24^, a semi-structured interview that assesses the severity of drug abuse problems and provides numerical scores for recent and lifetime drug use history. Cocaine craving was assessed using the Cocaine Craving Questionnaire^25^. Additionally, iCUD were asked to report the number of days since their last cocaine use. Verbal cognitive performance was measured with the Wide Range Achievement Test^25^ and nonverbal cognitive performance with the WASI Matrix Reasoning subtest^26^.

#### 2.2.3 Social functioning measures

A variety of measures were used to assess social functioning. The Experiences in Close Relationships Scale - Short Form (ECRS)^27^ assesses adult attachment style on two dimensions: anxiety and avoidance. Here, we used the total score, which combines both dimensions. The Social Network Index (SNI)^28^, which assesses the number of regular social contacts across 12 types of relationships (e.g., neighbors, spouse, relatives, friends), was used to determine social network size (i.e., the total number of individuals with whom the participant has regular contact). The Liebowitz Social Anxiety Scale (LSAS)^29^ was used to assess fear and avoidance of various social situations, with the total score reflecting the overall degree of social anxiety. The Resilience Scale for Adults (RSA)^30^ was used to assess the presence of protective factors that promote resilience (e.g., social support systems, psychological attributes). The Difficulties in Emotion regulation - Short Form (DERS;^31^ was used to measure the ability to regulate emotions effectively (i.e., recognizing, understanding, and accepting one’s emotional experiences and appropriately adjusting responses based on the situation).

### 2.3 Mentalizing neuroimaging task

To study the neural underpinnings of mentalization, we used the validated Why/How fMRI task^32^. This task features a 2 (social vs. factual inference) × 2 (face vs. hand stimuli) factorial design, with 4 blocks per condition. Each block starts with a question and includes 8 photographs depicting naturalistic human behaviors. Each photograph features either a familiar hand action or facial expression, preceded by a probe asking participants ‘why’ versus ‘how’ the behavior is being performed (yes/no responses). Each image appears twice in the task: once as the object of a social inference question probing the intention/mental state behind the behavior (i.e., ‘why’) and once as the object of a factual inference examining the behavior itself (i.e., ‘how’) for example, the emotional expression of selfdoubt versus the physical action of gazing down. Prior to performing the Why/How task, participants were told they would be performing a Photograph Judgment Test’ in which they would answer yes/no questions about photographs of people. Participants were also told they would have a limited amount of time to respond to each photograph and should make their best guess if they were uncertain about the answer. The total runtime of the task was 5 minutes. For data analysis, we combined the behavior type conditions (i.e., face vs hand stimuli) and focused only on the ‘why’ and ‘how’ conditions, following the approach of Spunt and Adolphs (2014). For localizing the mentalizing network, the contrast of interest is ‘why > how’.

### 2.4 MRI data acquisition

MRI data were acquired on a 3 tesla Siemens Skyra system (Siemens Healthcare, Erlangen, Germany) using a 32-channel head coil. Anatomical images were acquired in 224 sagittal slices using a T1-weighted sequence with the following parameters: TE = 2.07 ms, TR = 2400 ms, voxel size = 0.8 mm isotropic, flip angle = 8°, FOV = 256 mm (AP) × 180 mm (RL) × 256 mm (FH). Functional images were acquired in 40 interleaved slices using a T2*-weighted multi-band multi-echo planar imaging sequence with the following parameters: TE1/TE2/TE3 = 10.8/28.68/46.56 ms, TR = 1500 ms, GRAPPA factor = 2, voxel size = 3.6 mm isotropic, multi-band factor = 2, flip angle = 82°, FOV = 230 mm (AP) × 30 mm (RL) × 144 mm (FH). After the fMRI acquisition, two EPI volumes with the same parameters but opposite phase encoding directions were acquired for distortion correction.

### 2.5 fMRI data pre-processing

Multi-echo fMRI data were corrected for distortion using reversed-phase encoded volumes with FSL’s top-up procedure^33^. The data were then pre-processed and de-noised using independent components analysis (ME-ICA) with the tool meica.py as distributed in the AFNI neuroimaging suite (v2.5 beta10), which applies echo-time dependent denoising of EPI data to remove scanner, physiological, and motion artefacts in native space and then normalizes the data to MNI space. Denoised timeseries were then used for first and second-level analysis.

### 2.6 fMRI data analysis

fMRI data were analyzed using the MATLAB (7.5; MathWorks, USA)-based software package Statistical Parametric Mapping (SPM12, Wellcome Centre for Human Neuroimaging, London, UK). The first-level general linear model (GLM) was based on a double-gamma hemodynamic response function (HRF) and included two regressors of interest corresponding to the ‘why’ and the ‘how’ conditions and modelling the period between the onset of the first photograph and the offset of the last photograph of each block. Three regressors of no interest were added, modelling the kind of behavior depicted in the photograph (expression vs. action), the total accuracy within each block, and the total duration of each self-paced block.

The image resulting from the ‘why > how’ contrast (representing mentalizing) of each participant was then entered into a second-level, random effects analysis using 2 (*Diagnosis*: HC vs. iCUD) by 2 (*Trauma:* low vs. high) between-subject design, with *sex*, *education*, and *accuracy at ‘how’ questions* as covariates. The resulting statistical map was family-wise error corrected for multiple comparisons at the cluster-level (p_FWE_ < .05).

Additionally, mean beta weights were extracted from each participant’s ‘why > how’ contrast for the two significant frontal clusters identified in the main effect of *Trauma*, and for the precuneus cluster identified in the *Diagnosis × Trauma* interaction (Table 2). These beta weights were then used in correlation analyses with measures of social functioning and accuracy at the mentalizing task.

### 2.7 Statistical analyses

Statistical analyses were conducted in Statistical Package for the Social Sciences (SPSS; version 29; SPSS Inc., Chicago, IL). The normality and homogeneity of the data were assessed using Kolmogorov-Smirnov test for normality and Levene’s test for homogeneity of variances, respectively. The significance level of all statistical tests was set at p < .05.

#### 2.7.1 Sample

Two participants had to be excluded from the sample for analyses due to technical issues, and 15 participants (i.e., 7 CUD and 8 HC; see Table S1 for demographic information) were excluded due to poor performance (accuracy < 80%) on the mentalizing task, consistent with standard practices to ensure participants were engaged (e.g.,^34,35^). The final sample consisted of 45 individuals with CUD (iCUD) and 34 healthy controls (HC) who were classified into two subgroups based on childhood trauma severity (low vs. high). Trauma severity was categorized using a median split across the entire final sample (median = 42; range = 25–101), following established procedures^2,36^. Total CTQ scores did not significantly differ between iCUD and HC (p = .41), iCUD-low and HC-low (p = .08), nor CUD-high and HC-high (p = .61). This resulted in four groups: iCUD-low (N = 22), iCUD-high (N = 23), HC-low (N = 18), and HC-high (N = 16). For comparison of demographic and substance use characteristics between the groups, see Table 1.

#### 2.7.2 Demographic and substance use data

The continuous demographic and substance use data were normally distributed within each group. To compare these data among the four groups - or two groups when not applicable to the healthy control population - we used ANOVAs or *t*-tests, provided that the assumption of homogeneity was met. In case of violation of the homogeneity assumption, and given the unequal sample sizes, we used the non-parametric Kruskal-Wallis test instead. The categorical data (i.e., sex and race) were compared using Chi-square tests. The results of these comparisons (Table 1) informed the selection of covariates for further statistical analyses.

#### 2.7.3 Social functioning measures

For each of the five social functioning measures, we conducted a two-way ANOVA with *Diagnosis* (CUD vs. HC) and *Trauma* (low vs. high) as between-subject factors, and *sex* and *education* as covariates. To correct for multiple comparisons, p-values were adjusted using a sequential Bonferroni correction^37^. Significant group differences in social functioning, as indicated by significant ANOVA results, were further assessed for associations with changes in BOLD signal during mentalization using Pearson correlations (for details on the fMRI variables included in the correlation analyses, see section 2.6).

#### 2.7.4 Task data

Behavioral task data were processed in Matlab (version 24.1; The Mathworks Inc, Natick, MA) and statistical analyses in SPSS. Since the data did not significantly deviate from a normal distribution and the variances of the four groups did not significantly differ from each other, we conducted two-ways analyses of variance (ANOVA) to examine differences in *accuracy* (i.e., percentage of correct responses) and *reaction time* on the task. The between-subject factors were *Trauma* (low vs. high) and *Diagnosis* (CUD vs. HC), with *sex* and *education* as covariates. Separate ANOVAs were conducted for ‘why’ and ‘how’ questions.

## 3. Results

### 3.1 Social functioning

Significant main effects of *Trauma* were observed on the Experience in Close Relationships Scale, Liebowitz Social Anxiety Scale, and Resilience Scale for Adults scores (Table 2). Individuals with high trauma reported higher levels of anxiety and/or avoidance in close relationships (F_(1,73)_ = 8.65, p < .05), greater social anxiety (F_(1,73)_ = 11.47, p < .01), and lower resilience (F_(1,73)_ = 21.35, p < .01) compared to those with low trauma. A significant main effect of *Diagnosis* was observed on the Experience in Close Relationships score, with iCUD reporting higher levels of anxiety and/or avoidance in close relationships than HC (F_(1,73)_ = 7.16, p = < .05). None of the *Diagnosis* × *Trauma* interactions reached significance, though there was a trend for Experience in Close Relationships (Table 2).

### 3.2 Why/How Task Behavioral Results

**Figure 1.**
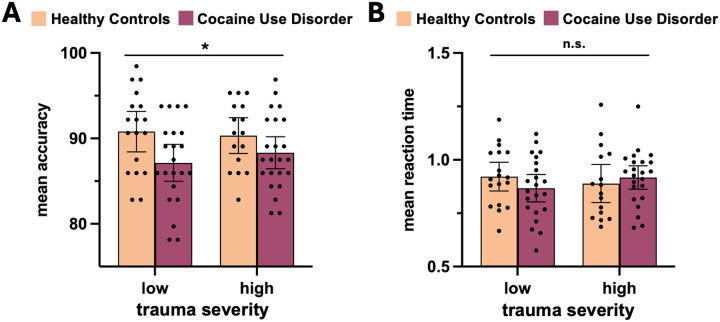
Behavioral performance on the mentalizing task. **A.** Mean accuracy on ‘why’ questions by group. **B.** Mean reaction time on ‘why’ questions by group.

HC performed better on the mentalizing task than individuals with CUD, as indicated by a significant main effect of *Diagnosis* on accuracy of responses to ‘why’ questions (F_(1, 73)_ = 4.46, *p* <.05) (Figure 1). Trauma severity was unrelated to accuracy, with no main effect of *Trauma* (F_(1, 73)_ = .21, *p* = .65) and no *Diagnosis* × *Trauma* interaction (F_(1, 73)_ = .3, *p* = .58). For ‘how’ questions, a main effect of *Trauma* showed that individuals with low trauma performed better than those with high trauma (F_(1, 73)_ = 8.85, *p* = <.01). There was no main effect of *Diagnosis* (F_(1, 73)_ = .77, *p* = .38) nor *Diagnosis* × *Trauma* interaction (F_(1, 73)_ = .91, *p* = .34). Reaction time (RT) analyses showed no significant main effects of *Diagnosis* (F_(1, 73)_ <= 2.93, *p* >= .09) or *Trauma* (F_(1, 73)_ <= .21, *p* >= .65), and no interaction (F_(1, 73)_ = 2.26, *p* >= .14) at neither ‘why’ nor ‘how’ questions.

### 3.3 Neuroimaging results

**Figure 2.**
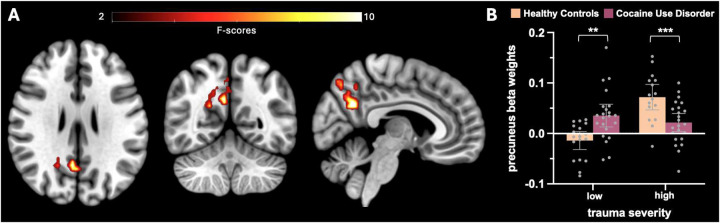
Interaction effect. **A.** Group-level random effects activation map for the ‘why > how’ contrast, depicting the *drug* x *trauma* interaction effect, corrected for multiple comparisons using a family-wise error correction at the cluster level (p_FWE_ < .05). **B.** Mean beta weights extracted from the significant cluster shown in A.

Whole-brain analyses showed a significant *Diagnosis* × *Trauma* interaction effect in the precuneus (BA 7; Figure 2A) for the ‘why > how’ mentalizing contrast. Post-hoc t-tests on the mean beta-weights extracted from this cluster showed that, within the low-trauma group, iCUD had greater activation compared to HC (t_38_ = −3.49, p < .01). In contrast, within the high-trauma group, iCUD had reduced activation relative to HC (t_38_ = 3.5, p < .001) (Figure 2B). Additionally, while activation did not differ significantly between iCUD with high versus low trauma (t_38_ = .99, p = .32), HC with high trauma had significantly greater activation than those with low trauma (t_38_ = −6.02, p < .001).

**Figure 3.**
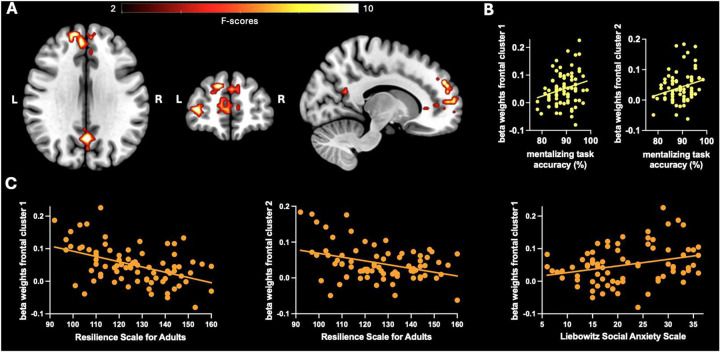
Main effect of trauma and correlations with mentalizing performance and social functioning. **A.** Group-level random effects activation map for the ‘why > how’ contrast, depicting the main effect of trauma, corrected for multiple comparisons using a family-wise error correction at the cluster level (p_FWE_ < .05). **B.** Correlations between activation in the frontal pole clusters shown in A and accuracy on the ‘why’ questions of the mentalizing task. **C.** Significant correlations between activation in the frontal pole clusters shown in A and measures of realworld social functioning.

Whole-brain analyses further showed a significant main effect of *Trauma*, where individuals in the high-trauma group had increased BOLD responses during mentalizing (‘why > how’) in the frontal pole [Brodmann Area (BA) 9 and 10] and the precuneus (BA 31) compared to those in the low-trauma group (BA 31; Figure 3A). No significant main effect of *Diagnosis* was observed. See Table 3 for further details on the peak activations.

### 3.4 Brain activation and social functioning

Extracted brain activation in the two frontal pole clusters (BA 9 and 10), which showed increased activity in high-trauma individuals (above), was positively correlated with mentalizing accuracy on the task (i.e., ‘why’ questions) (r_(39)_ >= .24, p < .05), and negatively correlated with resilience (r_(39)_ <= −.37, p < .001). Additionally, activation in one of these clusters (i.e., BA 9) was positively correlated with social anxiety (r_(79)_ = .29, p = .01). No other significant relationships were observed.

## 4. Discussion

This study aimed to identify novel relationships between childhood trauma, CUD diagnosis, and mentalizing-related brain function and behavior. Individuals with CUD performed worse than healthy controls in mentalizing behavior (i.e., inferring why particular social behaviors are being performed). A robust interaction effect between childhood trauma and CUD was observed with a distinct pattern of brain responses during mentalizing in the precuneus. Additionally, greater trauma severity was independently associated with increased activation in frontal regions, and activity in these regions was related to real-world social functioning - highlighting the potential clinical relevance of these neural differences.

The behavioral results showed that individuals with CUD performed worse than HC on the mentalizing task, corroborating previous findings that cocaine use disorder is associated with impairments in mentalizing^11,12^. In contrast, we did not observe a significant effect of trauma severity on task performance. This divergence from previous studies reporting trauma-related mentalizing deficits may reflect differences in the types of trauma experienced^38^ or in the type of mentalizing task used, particularly since the Why/How task was optimized as a functional localizer rather than to assess behavior^32^.

Notably, trauma severity was associated with significant differences at the neural level. Specifically, individuals with higher trauma exposure showed increased activation in frontal pole regions during mentalizing. These areas have been shown to be involved in mentalizing about the self and others^32,39^ and regulating social-emotional function^40^. Activity in these regions during the mentalizing task was positively correlated with task accuracy, suggesting that increased activation may support performance and help individuals with high childhood trauma maintain accuracy at levels comparable to those with low trauma. Importantly, greater activation in these same regions was also associated with lower resilience and higher levels of social anxiety in daily life. Thus, this pattern may reflect a compensatory neural mechanism that facilitates performance on simple mentalizing tasks in high trauma individuals yet may contribute to broader difficulties in social and emotional functioning.

We observed a significant interaction between childhood trauma severity and CUD in the precuneus, a core hub of the default mode network (DMN)^41^ and a key region of the mentalizing network^32,39^ involved in mental imagery^42^ and self-referential thinking^43^. Specifically, individuals with CUD and low trauma showed greater activation than HC with low trauma, whereas individuals with CUD and high trauma showed reduced activation compared to HC with high trauma. Notably, there were no significant differences in precuneus activation between iCUD with low versus high trauma, while HC showed a marked increase in activation with higher trauma severity. This pattern suggests that childhood trauma may modulate precuneus responses to mentalizing in healthy individuals, potentially reflecting increased engagement or compensatory effort, consistent with prior findings that precuneus activity increases with task difficulty^44,45^. In contrast, this trauma-related modulation appears blunted or absent in individuals with CUD. One interpretation is that CUD may interfere with adaptive neural responses to early adversity during mentalizing, consistent with evidence that substance use disorder is associated with increased DMN recruitment during drug cue exposure but reduced activation during non-drug related social-emotional processing^46^, and that CUD disrupts both connectivity within the DMN and its interaction with other networks^47–49^. Alternatively, it is also possible that CUD dampens excessive activation in high-trauma individuals, potentially normalizing hyperactive precuneus responses often observed in trauma-exposed populations^19,50,51^. Given that precuneus activation was not significantly associated with real-world social functioning measured in this sample, the functional significance of this interaction warrants further investigation.

This study provides new insights into the neural mechanisms underlying social cognitive functioning in individuals with cocaine use disorder and/or a history of childhood trauma. By studying the distinct and interactive effects of trauma and substance use on mentalizing-related brain activity and behavior, our findings enhance understanding of social dysfunction in these highly co-morbid conditions and open avenues for future research aimed at identifying targets for intervention.

### 4.1 Conclusion

This study reveals how childhood trauma and cocaine use disorder influence brain activity related to mentalizing, with potential implications for social functioning in daily life. Greater trauma severity was associated with increased frontal activation, which may support task accuracy but is linked to poorer real-world social functioning. Additionally, CUD appears to modulate the relationship between trauma severity and precuneus activity during mentalizing, providing neural insights into how trauma history and CUD interact.

## Supplementary Material

Supplementary Files

This is a list of supplementary files associated with this preprint. Click to download.

- Table1.docx
- Table3.docx
- Table2.docx
- Supplement.docx

## Figures and Tables

**Table 1.** Demographics, substance use characteristics, and Childhood Trauma Questionnaire (CTQ) score by group.

Demographics	HC-low (n = 18)	HC-high (n = 16)	iCUD-low (n = 22)	iCUD-high (n = 23)	comparison
Age	44.4 (9.9)	45.78 (10)	46.63 (10.9)	45.19 (9.1)	n.s.
Sex (F/M)	10/8	10/6	5/17	5/18	^+^p < .05
Race (aa/white/other)	12/4/2	7/7/2	9/9/4	15/8/0	^+^n.s.
Education (years)	15.44 (2.3)	14.63 (2.1)	13.16 (1.3)	13.54 (2.1)	p < .01
Verbal cog. perf.	105.11 (10)	105.06 (10.6)	104 (10.7)	106.91 (8.5)	n.s.
Nonverbal cog. perf.	11.72 (2.3)	10.63 (2.4)	9.77 (2.9)	11.35 (2.3)	n.s.
					
**Substance use**					
Cocaine UTOX on MRI day (pos/neg)	n/a	n/a	10/12	9/14	^+^n.s.
Craving for cocaine	n/a	n/a	1.36 (1)	1.51 (1.2)	n.s.
Recency of cocaine use (days)	n/a	n/a	3.04 (2.7)	15.56 (31.3)	*p < .05
Lifetime use (years):					
Nicotine	3.61 (9.8)	1.75 (5.8)	17.8 (11.9)	21.48 (12)	*p < .001
Alcohol	14.8 (14.6)	13.66 (13.37)	24.29 (11)	21.65 (11)	p < .05
Cannabis	0.5 (1)	3.94 (9.1)	9.27 (10.5)	11.46 (13.3)	*p < .01
Cocaine	n/a	n/a	20.36 (11.2)	17.76 (11.5) ‡	n.s.
					
**CTQ score**	30 (4.8)	58.38 (13.7)	32.14 (4.6)	62.04 (13.8)	

HC-low = healthy controls with low trauma, HC-high = healthy controls with high trauma, iCUD-low = individuals with cocaine use disorder and low trauma, iCUD-high = individuals with cocaine use disorder and high trauma.

‡ N = 22 for this variable due to outlier removal. Group comparisons were conducted using various statistical tests:

* indicates the Kruskal-Wallis test,

+ indicates the Chi-square test, and unmarked values represent results from an ANOVA or t-test.

**Table 2.** Social functioning outcomes.

	Scores				Statistics			
	HC low	HC high	CUD low	CUD high		F	uncorrected p-value	S-B corrected p-value
**Experiences in Close Relationships Scale**	29.17 (10.1)	40.31 (10.3)	40.05 (8.7)	42.57 (11)				
					main *Trauma* effect	8.65	.004	.012
					main *Diagnosis* effect	7.161	.009	.045
					*Trauma* × *Diagnosis* interaction	3.412	.069	.345
**Social Network Index**	10.67 (8.6)	8.94 (4.9)	9.59 (6.1)	9.7 (6.9)				
					main *Trauma* effect	.308	.58	.58
					main *Diagnosis* effect	0.72	.789	.789
					*Trauma* × *Diagnosis* interaction	.389	.535	1.605
**Resilience Scale for Adults**	135.28 (15.9)	118.38 (17.63)	132.41 (12.5)	118.3 (15.3)				
					main *Trauma* effect	21.346	< .001	< .001
					main *Diagnosis* effect	3.238	.076	.228
					*Trauma* × *Diagnosis* interaction	.328	.568	1.136
**Liebowitz Social Anxiety Scale**	15.28 (6)	23.06 (6.8)	21.64 (8)	25.65 (8.4)				
					main *Trauma* effect	11.467	.001	.004
					main *Diagnosis* effect	5.978	.017	.068
					*Trauma* × *Diagnosis* interaction	.988	.324	1.296
**Difficulties in Emotion Regulation Scale**	27.67 (12.1)	33.13 (10.1)	30.55 (10.4)	36.53 (13)				
					main *Trauma* effect	4.452	.038	.076
					main *Diagnosis* effect	1.7	.196	.392
					*Trauma* × *Diagnosis* interaction	0.41	.839	.839

HC low = healthy controls with low trauma, HC high = healthy controls with high trauma, iCUD low = individuals with cocaine use disorder and low trauma, and iCUD high = individuals with cocaine use disorder and high trauma, S-B = Sequential-Bonferroni.

**Table 3.** Mentalizing neuroimaging results. Activation clusters and corresponding size, anatomical region, FWE-corrected p-value, peak coordinate in MNI space, and z-score for the main effect of trauma and drug x trauma interaction effect on the ‘why > how’ contrast, FWE-corrected using a cluster significance level of p < .05. Reported anatomical labels were determined using the Harvard-Oxford cortical structural atlas^52^ and correspond to the location of maxima within each cluster.

				peak coordinate			
cluster	region	# voxels	PFWE-corr	x	y	z	Z_E_
**Main effect of trauma**							
1	Frontal pole (BA 9)	86	.007	13	53	31	3.77
2	Frontal pole (BA 10)	135	<.001	−9	64	16	3.73
3	Precuneus (BA 31)	129	<.001	−2	−62	27	3.87
							
**Interaction effect**							
1	Precuneus (BA 7)	88	.006	2	−69	49	3.55
